# Supplementary material for: Patterns of Intron Gain and Loss in Fungi
Source: PLoS Biol. 2004 Nov 30;2(12):e422. doi: 10.1371/journal.pbio.0020422 (PMC532390; doi:10.1371/journal.pbio.0020422)
Supplement: Table S1 — Also available at http://genes.mit.edu/NielsenEtAl/. (4.3 MB ZIP). [file pbio.0020422.st001.zip › NielsenEtAl/html/1210.html]

AN5139.1.NCU08484.1.MG06969.1.FG10869.1


```
 CLUSTAL W (1.82) Multiple Sequence Alignments - Introns Inserted


Sequence 1: MG06969.1	854 aa
Sequence 2: FG10869.1	854 aa
Sequence 3: NCU08484.1	879 aa
Sequence 4: AN5139.1	1207 aa
Alignment Length: 1280 aa
Number Identitical Residues: 369 aa
Alignment Score (without introns) 20471


MG06969.1 	---MASRRYVPRQGH~TEGTTHCAYTPNGSQLVTVGSNNTIRCYKTGSDGEPKNIDDCGE
NCU08484.1	MTTLIASRPRSRPAH~PPGATYCAYTPDGTRLVTVGSNNTTRLYKTGYEGEPTNIDDCQE
FG10869.1 	MDSTIAPKPRPRPAH~TQGTTRCAYTPDGNRLVTVGSNNTIRLYKTGSDGEPINIDDCQE
AN5139.1  	--MSDPVRPRGRPAH1TPGTTVLTYTPDGRYIITGGSNSAIRIYTDGEDGEPKTVEEGAD
          	     . :   * .* . *:*  :***:*  ::* ***.: * *. * :*** .:::  :

MG06969.1 	QNVAVAAT~DRFFVVGSEDGTVSLFSLETHSFETYLLRTGLPIRDIALSSDGKWCAVAGD
NCU08484.1	QNLALATS~NNYFVAGSEDGTVSLYSLDTNAFDRFLLRTSLPVRDVAISPDHQWCAVASD
FG10869.1 	QNMTVAAG~DQFFVVGSEDGTVSLYSLETNIFERFLTRTTLPIRDVALTADNQWCAVASD
AN5139.1  	AHLAIGAT0NEYFFMGAEDGTVWQYEVKSGRMDKLLTRTALAVRDIAITKDNGWVAVASD
          	 ::::.:  :.:*. *:*****  :.:.:  ::  * ** *.:**:*:: *  * ***.*

MG06969.1 	~ELTVKIVSTTDNTVVRHAREHGRPTKHVSFDPKGSTLTLSCTDGAIYIYSLTSEQPELI
NCU08484.1	~ELTVRLVGVHDNTQLRTLKEHGKPTKHLTFDPKGSLLALSCTDGVIYIYSLTAEHPELI
FG10869.1 	~ELSVKIVNTKDISQVKHLREHARAVRNVSFDPQGRLVALSGTDGTVYVYSLTAEEPELI
AN5139.1  	2ELTVKLVNIEDMTKVKYMREQTKGTKHITFDPNGRYVAVSCTDGIVYLYSMDTEEPELA
          	 **:*::*.  * : ::  :*: : .::::***:*  :::* *** :*:**: :*.*** 

MG06969.1 	RKIDGILGSLEMESEASGRVVWHPDGRAFAVPTPTRDIEVVSKNDWEKQRVFANGHEGDI
NCU08484.1	RKVDGVIGRLETESEASSRAVWHPDGRAFAAPTPTRDIQVISKNDWEKQRVFKSGHDGDI
FG10869.1 	RKVEGVIGVVDAESEKSTRVAWHPDGRAFAVPTPMRDIQVISKNDWEKQRTFANGHLSDI
AN5139.1  	RKLDGVIRRLEPEDEATARVVWHPDGTAFATADASRDIALFSVGEWKKEMSFSGGHNGDI
          	**::*::  :: *.* : *..***** ***.. . *** :.* .:*:*:  * .** .**

MG06969.1 	TAMAWSPNGSMIATAAKDGKLLIWESRGQSVIARHSYANIMDIVWHPKANIVSFTTFAGE
NCU08484.1	TALSWSPNGALLASAGKDKKLLIWSTKDQSVIARYEYPNVTDICWHPTKNLASFTTSDGE
FG10869.1 	TALAWSPNGAMLASASKDNKVLIWETRTQSVIARYDYPNVIDLAWHPTKNIISFTTTDGE
AN5139.1  	TAMSWSPNGALMVTAAKDGQVLLWESKTQKILHRYNFPNVINLAWHPTKNGVSLTTSDGE
          	**::*****:::.:*.** ::*:*.:: *.:: *:.:.*: :: ***. *  *:**  **

MG06969.1 	VFICADFVTADFTPLLQLPLQASPFIHDPLAEVSVNARRPAINGSKE--IPSRPRRDSLN
NCU08484.1	LYISPDFVPEQFASLLRLPKQPAPFIHDPLTELSNNTRRLEVSGPKP---DARPRRDSVD
FG10869.1 	VYIYPDFLTDQFSPLLKLITQPAPFIHDPLAEISANRRPPPQNGQKQHGLPTRPRRDSLG
AN5139.1  	IFIFDGFVPKDYQALLQKPLQAAPIFPGALTEISDNVQRPLASRPKE-----ALRRGSID
          	::*  .*:. :: .**:   *.:*:: ..*:*:* * :    .  *        **.*:.

MG06969.1 	S-LDSFA-GGIPEDEDDFVVDDDGAGYTLNSN--GKRPYDGTGVGE-HANKR--PNMLRP
NCU08484.1	ADLDDLLDENYG-DDDDFVVDDDGAGYTLNAG--RKRPAEDDAIYESHTAKR--GNFVQP
FG10869.1 	S-LDSFLEGGEGYGDDDFVEDDDGAGYTAGLG--QKRGRDDDDGLG-FANKR--RHLLEP
AN5139.1  	S-LDDIL--GYDQDMEDFVEDDDGAGYVEDVNGFGKRTNKHLGDIEGHMDKRTLTSFPKP
          	: **.:   .   . :*** *******. . ..  **  .      ..  **:   : .*

MG06969.1 	QYHEAFQPGSTPWRGNRKYLC~LNLTGFVWTVDQDSHHTVTVEFYDHELHRDFHFTDTFL
NCU08484.1	QHHPSFQPGSTPWRGNRKYLC~LNLIGFVWTVDQDSHNTVTVEFYDHEFHRDFHFTDTFL
FG10869.1 	QYHESFQPGATPWRGNRKYLC~LNLIGFVWTVDQDGHHTVTVEFYDHEFHRDFHFTDTFL
AN5139.1  	KIHPPLQPGSTPWRGNRRYLC1LNLTGAVWTVDQETHNTVTVEFYDRELHRDFHFTDPFL
          	: * .:***:*******:*** *** * ******: *:********:*:********.**

MG06969.1 	YDKACLS~EHGTLFSCPPRDD-KPAVLFYRPHETWTQRSDWRTQLPAGEAVLAMSLSDSF
NCU08484.1	YDKACLN~DKGSLFSCPPKEDGSPAVVFYRPHETWTQRNDWRIQLPKGEAVLAMSLSDSY
FG10869.1 	YDKACLT~EHGTLFSCPPKDD-APAVIFYRPHETWTQRSDWRTELPRGEVVTAMSLSESF
AN5139.1  	YDRACLI1DD------------SPATIFYRPHETWTTRADWKTTLPKGEHIR--------
          	**:***  :.             **.:********* * **:  ** ** :         

MG06969.1 	VTVT~TTAGYVRVFTLFGVPYRVYRPKSFPMVTCASWKDYVLTMGNGPVGADGNT-RLLY
NCU08484.1	ITVT~TTGNYVRIYTLFGIPVRVYRPKSTPMVTCASWRDYVLTMGNGPVGADGMS-RLLY
FG10869.1 	ITVT~TSANYVRVFTLFGMPYRVYRPKSTPMVTCASWRDYVITMGNGPMGADGNT-RLLY
AN5139.1  	---V1TTKDYVRVYTLYGTPFRVYRQKS-PAVTCAAWRDYVMTIGNGPLGSDGRTGTLRY
          	   . *: .***::**:* * **** ** * ****:*:***:*:****:*:** :. * *

MG06969.1 	TIEDVKRDEICQNEDTVALPEGATVKSVFFSDDG0DPCIYDSTGTLLTLLHWRQPSRASW
NCU08484.1	TIENIKRDEVCQNEDTVALPEGATVKSVFFSDNG0DPCIYDSTGTLLTLLHWRRPSRAVW
FG10869.1 	TIENVKRDEICQNEDTVALPEGATLKSVFFSDNG0DPCIYDSTGTLLTLLHWRQPSRASW
AN5139.1  	SIENVKRDEICQNEDVVAIPEGAELKSVFFTDMG0DPCIYDTEGVLLVLQHWRTQGQARW
          	:**::****:*****.**:**** :*****:* * ******: *.**.* ***  .:* *

MG06969.1 	VPLLDTKLLSRLASGRKHESYYPIAVADHKFHCIILKGGDQYPYFPRPLLSEFEFSVPLA
NCU08484.1	VPLLDTRLLPRLASGRKKESYFPIAVADHKFHCIILKGGDQYPYFPRPLLSEFDFAVPLT
FG10869.1 	VPLLDTKLMDRLASGRKNESYFPIAVADNKFHCIILKGGDQYPYFPRPLLSEFDFSIPIS
AN5139.1  	VPLLDTKQLERLASGRKEETYWPVAVAQDKFHCIILKGGDRYPYFPRPMLSEFDFQIPIS
          	******: : *******.*:*:*:***:.***********:*******:****:* :*::

MG06969.1 	SAP--SGSKKKKQLNS---DGADGSEDEE------EEDEG-----------AETQKLDQQ
NCU08484.1	SAKPKKQSKKKKTRSGDDSMDVDGEDEEDNDFSASEADDSDSENNADPLTTKESLPLTQS
FG10869.1 	SAP--KPSKRKVREGSEDLDMGDGDDDKD------EEEDGS----------SETRKLEQQ
AN5139.1  	DKP-----KPQE-------EDEEGSTARN-----------------------DSMRLEES
          	.       * :           :*.  .:                       ::  * :.

MG06969.1 	FLLRSIRAAQVLDVLESSSTSKSVSRAQQQSLARLELEIDKTLLQMLAVECREGEERGMR
NCU08484.1	FLLSSLRAAQLSDMLESSRS----TASQRAALARLELDIDKTLLQLLAIECREGEDRGMR
FG10869.1 	FMLHGVKAAQLRDLVEATSG----SHNQRSQLSRLELEIDKTLLQLLAVECREGEERGMR
AN5139.1  	FVRGNLLLSLFQDLLSATHA----TATQRAELSRKELELDKVLLQLLAIECREGEERGMK
          	*:  .:  : . *::.::      :  *:  *:* **::**.***:**:******:***:

MG06969.1 	ALEMVSLMRDRSGRMIEAACKVAERYGRTILGDKIRELGEKM~VRGEG------------
NCU08484.1	ALEVVKLMKDRTGRMIEAAGKVAERYGRRILGEKIREVGERR~VAGED------------
FG10869.1 	ALEMVQLMRDRTGRMMEAASKVAERYGRTILGDKIREVGEKR~VEGLG------------
AN5139.1  	ALELVHMMKDRNGKMVEAAVKVAQRYNRGVLEDKIRDLAARR2HSTATGLWVNVIISSIF
          	***:* :*:**.*:*:*** ***:**.* :* :***::. :       .   .   ::  

MG06969.1 	-------------DDDMDEDDEF-------------------------------------
NCU08484.1	-------------ESEGEEEEDPFA-----------------------------------
FG10869.1 	-------------DDDF-------------------------------------------
AN5139.1  	EFPFSKFQFFNLQLSDNEYSNALITPLSSPAMPKFALSRSSTFASSRTTRFYRFRSNSSR
          	. . :. .  . . .: . ..   :. ::.: .. : : ::: ::: ::      :.:: 

MG06969.1 	------------------------------------------------------------
NCU08484.1	------------------------------------------------------------
FG10869.1 	------------------------------------------------------------
AN5139.1  	CASSHSQIRHDIPRSPSPQAHLPLLPSQSGPGFSFKYSLTDDLTPPNDHKPPDERIIKLG
          	.::: :.   . . :.:..:  .  .:.:... : . : :.. :.... .....   . .

MG06969.1 	--~---------------------------------------------------------
NCU08484.1	--~---------------------------------------------------------
FG10869.1 	--~---------------------------------------------------------
AN5139.1  	KT1LRILSPLLPTILTNPLPPHILSPNVTLHLFPSTHPHLPTVKGRTLYRAALWAVPVAW
          	.:     :.  .:  :.. ..   :.. :    .:: .  .: .. :   ::  : . : 

MG06969.1 	------------------------------------------------------------
NCU08484.1	------------------------------------------------------------
FG10869.1 	------------------------------------------------------------
AN5139.1  	SSVPLVGNVKLQILSERIVRGGIVPDPQVDTAHGTQAKNGDCGDERLVVRWRTEKPNSHS
          	:: .  .. . .  :.    ..  .... .:: .:.:........       :....: :

MG06969.1 	------------------------------------------------------------
NCU08484.1	------------------------------------------------------------
FG10869.1 	------------------------------------------------------------
AN5139.1  	SSSSSKSETSTLSTASSSPLKRDPKTGDESKTNTNKGLSVLLGGDAPIFKLSRDDQFTGL
          	:::::.:.::: ::::::. . ...:...:.:.:... :   ...:.  . : ... :. 

MG06969.1 	------------------------------------------------------------
NCU08484.1	------------------------------------------------------------
FG10869.1 	------------------------------------------------------------
AN5139.1  	FIFSFDEEGRVLTQTIEHAEDAGGWERTAKFVTLTDWLIGKARGSLDTPQPAGLCCSGTG
          	   : ....   :.: . :..:.. . ::.  : :.   ..: .: .:...:. ..:.:.

MG06969.1 	-----------------------------
NCU08484.1	-----------------------------
FG10869.1 	-----------------------------
AN5139.1  	QDPGHGREGRWRSDEAEKIRISGASGSSL
          	.... . ..   :..:..   :.::.::
```
